# Enhanced self-renewal of human pluripotent stem cells by simulated microgravity

**DOI:** 10.1038/s41526-022-00209-4

**Published:** 2022-07-04

**Authors:** S. Timilsina, T. Kirsch-Mangu, S. Werth, B. Shepard, T. Ma, L. G. Villa-Diaz

**Affiliations:** 1 Department of Biological Sciences, Oakland University, Rochester, MI 48309 USA; 2 Department of Computer Science, Engineering, Oakland University, Rochester, MI 48309 USA; 3 Department of Bioengineering, Oakland University, Rochester, MI 48309 USA

**Keywords:** Cell biology, Developmental biology, Environmental sciences

## Abstract

A systematic study on the biological effects of simulated microgravity (sµg) on human pluripotent stem cells (hPSC) is still lacking. Here, we used a fast-rotating 2-D clinostat to investigate the sµg effect on proliferation, self-renewal, and cell cycle regulation of hPSCs. We observed significant upregulation of protein translation of pluripotent transcription factors in hPSC cultured in sµg compared to cells cultured in 1g conditions. In addition to a significant increase in expression of telomere elongation genes. Differentiation experiments showed that hPSC cultured in sµg condition were less susceptible to differentiation compared to cells in 1g conditions. These results suggest that sµg enhances hPSC self-renewal. Our study revealed that sµg enhanced the cell proliferation of hPSCs by regulating the expression of cell cycle-associated kinases. RNA-seq analysis indicated that in sµg condition the expression of differentiation and development pathways are downregulated, while multiple components of the ubiquitin proteasome system are upregulated, contributing to an enhanced self-renewal of hPSCs. These effects of sµg were not replicated in human fibroblasts. Taken together, our results highlight pathways and mechanisms in hPSCs vulnerable to microgravity that imposes significant impacts on human health and performance, physiology, and cellular and molecular processes.

## Introduction

Microgravity contributes to the challenging environment of space that causes several pivotal alterations in living systems. The possibility of simulating microgravity by ground-based systems provides research opportunities that will lead to a better understanding of the in-vitro biological effects of microgravity on cells, while eliminating the challenges inherent to spaceflight experiments, including limited availability, high cost, and complexity of experimental conditions^1–3^. Stem cells are one of the most prominent cell types to study, due to their self-renewal and differentiation capabilities that maintain homeostasis in the body. In essence, pluripotent stem cells (PSCs) of human origin (human embryonic stem cells: hESCs, and human-induced pluripotent stem cells: hiPSCs) are of particular interest for their capacity to differentiate into all cells of the body, and for their potential use in personalized and regenerative medicine^4–7^.

Although considerable progress has been made in identifying the molecular mechanisms regulating the biological functions of hPSCs, it remains unknown whether such mechanisms will be altered by microgravity. Studies using simulated microgravity (sµg) have improved our knowledge on the effects of microgravity regarding morphology, migration, proliferation, and differentiation of multiple stem cell populations. In general, these studies have demonstrated that under sµg conditions stem cells have reduced capacity for differentiation^8–10^, while their self-renewal is enhanced^8,9,11–13^. In addition, it has been documented that during spaceflight diverse physiological conditions are affected, including loss of muscle mass^14–16^, compromised immune system^17,18^, susceptibility to ocular cataracts^19^, loss of bone density^20–22^, cardiac stress^23,24^, among others, indicating that tissue regeneration is affected, and this might be due to altered stem cell behavior. All of these findings indicate that the study of stem cells under sµg conditions can shed light on potentially new molecular mechanisms involved in self-renewal and differentiation.

We developed a fast-rotating 2-D clinostat to study the biological effects of sµg on hPSCs. In our method, hPSCs are cultured adherent to Matrigel-coated surfaces and with xeno-free and chemically defined medium, conditions well-characterized that support their self-renewal^25^. Our clinostat spins with an axis of rotation that is perpendicular to the gravitational vector, which averages the forces acting on the cells to near-zero^26–28^. Data from this study indicate that several physiological processes of hPSCs, including self-renewal, telomere maintenance, differentiation, proliferation, and cell cycle regulation are influenced by sµg. Based on these findings, we attempt to build a model to better understand the molecular mechanisms behind the regulation of hPSCs.

## Methods

### Preparation of chamber slides and cell culture substrate

**Fig. 1:**
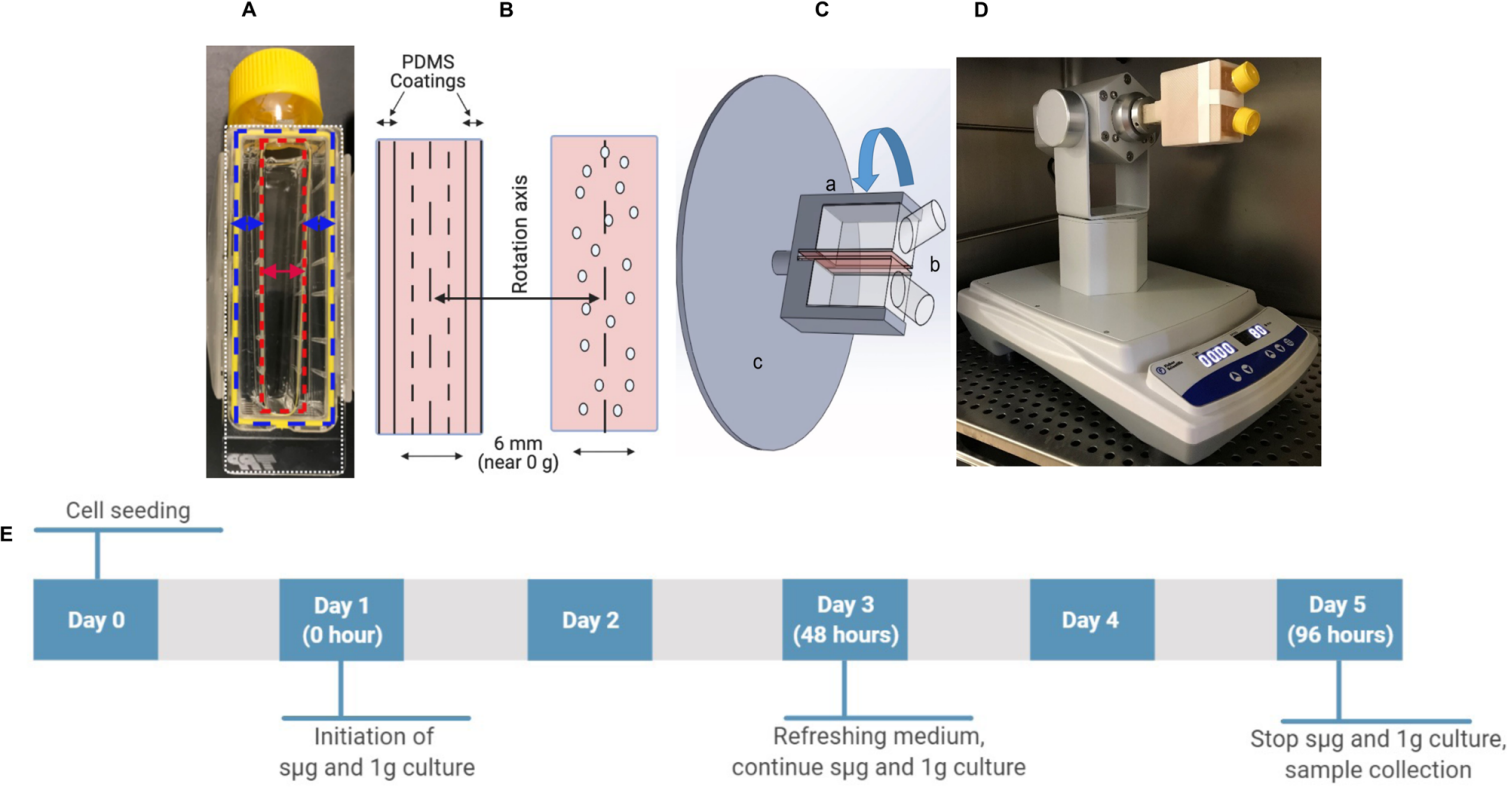
Experimental setup to simulate microgravity. **A** Clipmax chamber-slide flask with a small cell culture channel created (represented by a red double-sided arrow) after filling the two sides (represented by two blue double-sided arrows) and the top of the chamber-slide flask (not shown) with PDMS. **B** Distribution of microgravity forces in relation to the rotation axis at the area where cells are cultured. Oval circles represent cells. **C** Illustration of the developed device to simulate microgravity: (**A**) indicates 3-D printed adapter connecting two cell culture flasks (**B**) to the spinning bolt of a sample rotator instrument. **C** Indicates the bottom surfaces of the flasks where cells are attached (illustrated in red), which are positioned back-to-back and located in the axis of rotation. **D** Our developed rotary cell culture system (RCCS) with 2 flasks affixed to the system and located inside of the cell culture incubator, ready to generate simulated microgravity. **E** Experimental design used in this project.

Chambers slides were prepared inside Clipmax chamber slides flasks (TPP Techno Plastic Products AG, Switzerland), in which a small culture channel was created with polydimethylsiloxane (PDMS), as described before^29^. Briefly, a 1:10 (w/w, curing agent: base monomer) ratio PDMS pre-polymer (Sylgard 184, Dow-Corning, Midland, MI) was poured over the two sides and on the top of the Clipmax chamber-slide flasks, and cured at room temperature for 12 h on each side, leaving a centered channel for cell culture at the bottom of the chamber without PDMS (Fig. 1A). The cell culture area has an approximate dimension of 10 × 60 mm and aligns to the rotation axis (Fig. 1B). Matrigel (BD BioSciences, San Jose, CA) was diluted to a concentration of 100 μg/ml in cold DMEM/F12 (Gibco Life Technologies, Waltham, MA) and it was applied to cover the cell culture area at the bottom of the chamber. The coating was allowed to polymerize during 2 h of incubation at room temperature^30^. Before plating cells, the excess of Matrigel-DMEM/F12 solution was aspirated, and chambers were washed with Dulbecco’s phosphate-buffered saline (D-PBS) (Gibco Life Technologies).

### Simulating microgravity

A fast-rotating 2-D clinostat was developed following a previous report to generate simulated microgravity (sµg) conditions for culturing adherent cells^27^. Two Clipmax chamber slides were placed back-to-back on a custom-designed holder that was 3-D printed using ABS plastic (Fig. 1C). The holder was connected to a Multi-purpose Tube Rotator (Fisherbrand, Ontario, Canada). The instrument holding the two cell culture chambers was placed inside of a dedicated cell culture incubator set up at 37 °C, 95% humidity, and 5% CO_2_ conditions (Fig. 1D). As a control, cells were cultured in cell culture chamber slides in static 1g conditions for the same period of time (Fig. 1E).

### Cell culture, evaluation of pluripotency, and induced differentiation of human pluripotent stem cells (hPSC)

All experiments were repeated at least in triplicates with NIH-approved hESC H1 and H9 (WA01 and WA09; WiCell Research Institute Inc., Madison, WI) and hiPSCs derived in our laboratory. The undifferentiated hPSCs were cultured on Matrigel-coated tissue culture plates (Applied Biosystems, Foster City, CA) with StemFlex Medium (Gibco Life Technologies) and maintained in cell culture incubators with high humidity and 5% CO_2_ at 37 °C. For experimental conditions in the chamber slides, hPSCs were dissociated into single cells using L7 dissociation solution (Lonza, Basel, Switzerland), and 10,000 cells were seeded on Matrigel-coated chamber slides with StemFlex medium and 10 μM of ROCK inhibitor (Stem Cell Technology, Vancouver, Canada)^31^. Twenty-four hours post-seeding, two chamber slides were transferred to sµg condition after completely filling with StemFlex medium, while the other remaining two chamber slides were assigned as the control group and cultured at 1g in static conditions. Forty-eight hours (h) later, the clinostat rotator was stopped for ~5 min to replace the culture medium, and immediately after the rotation was resumed. The cells were further cultured for a total of 96 h under both experimental and control conditions, as cell confluency was reached and cellular responses to culture conditions were clearly evident. Human foreskin fibroblasts (hFF-1; ATCC) were cultured in similar conditions with MEM Alpha medium supplemented with 10% fetal bovine serum (FBS). Immediately after stopping the clinostat at the end of the cell culture experiment, the cells were processed for subsequent studies.

In-vitro analysis of pluripotency of hPSCs from each group was evaluated by embryoid bodies (EB) formation. Cells were cultured in suspension with MEM Alpha (Gibco Life Technologies) supplemented with 10% FBS for 10 days to make EBs. Direct differentiation of hPSCs was performed on the sµg chamber slides with chemically defined medium (CDM) consisting of DMEM/F12 (Gibco Life Technologies) supplemented with 1 × N2 (Invitrogen), 1 × B27 (Invitrogen), 0.11 mM 2-mercaptoethanol, 1 mM nonessential amino acids (Gibco Life Technologies), 2 mM l-glutamine (Gibco Life Technologies), and 0.5 mg/ml bovine serum albumin (BSA) (fraction V; Sigma-Aldrich) for 4 days following established protocols^32^. To induce trophectoderm and neuroectoderm differentiation, cells were cultured in CDM supplemented with 50 ng/ml human recombinant bone morphogenetic protein (BMP)-4 (Stemgent, Cambridge, MA) and 4.5 μM retinoic acid (RA) (Stemgent), respectively.

### Quantitative analysis of undifferentiated colony size and the total number of cells

Microscopic images of undifferentiated hPSC colonies were used to calculate the colony area of at least 10 randomly selected colonies after 96 h of culture under sµg and 1g conditions using NIH ImageJ software (http://rsb.nih.gov/ij). Data from independent replicates were averaged and standard deviations were calculated, compared, and used for statistical analysis. The total number of cells after 96 h of culture under sµg and 1g conditions was calculated after the dissociation of colonies into single cells and counted using a hemocytometer.

### Immunofluorescence staining

Immediately after stopping the clinostat rotator cells were fixed with 4% paraformaldehyde (Electron Microscopy Sciences, Hatfield, PA) for 10 min, permeabilized with 0.1% Triton X-100 (Roche Applied Science, Indianapolis, IN) for 10 min, incubated in TBS with 0.1% sodium borohydride for 5 min and incubated in blocking solution (1% BSA/PBS and 10% normal donkey serum) for 1 h, all at room temperature (RT). Then samples were incubated overnight at 4 °C with primary antibodies diluted in 1% BSA and 1% normal donkey serum. The next day samples were washed three times with PBS, followed by 1 h of exposure to secondary antibodies diluted in 1% BSA and 1% normal donkey serum at RT. Samples were then incubated for 10 min with DAPI, followed by three wash steps with PBS. These steps were performed at RT and in dark conditions. Samples were treated with BD Stabilizing Fixative solution (BD Biosciences) diluted in PBS for 5 min, then treated with ProLong Gold Antifade Reagent (Molecular Probes Life Technologies, Grand Island, NY), and mounted with a glass cover slide. Sample images were captured using an EVO FL M5000 cell imaging system (ThermoFisher Scientific). The following antibodies were used: OCT4 (SC8629, Santa Cruz Biotechnology, Dallas, TX), NANOG (MABD24, Millipore, Billerica, MA), SOX2 (AB5603, Millipore), integrin α6 (MAB1378, Millipore, Billerica, MA), integrin β1 (MAB1959, Millipore), SSEA-4 (MAB4304, Millipore), TRA-1-60 (MAB4360, Millipore), and TRA-1-81 (MAB4381, Millipore). The mean fluorescent intensity was calculated using NIH ImageJ software.

### Western blot analysis

The following antibodies were used: OCT4 (SC8629, Santa Cruz Biotechnology), NANOG (MABD24, Millipore), SOX2 (AB5603, Millipore), Integrin α6 (MAB1378, Millipore), Integrin β1 (MAB1959, Millipore), PSMD11 (NBP2-59484, Novus Biologicals; Centennial, CO) and GAPDH (2118, Cell Signaling Technology). Whole-cell lysates were prepared from cells, separated on 10% SDS-polyacrylamide gel, and transferred to polyvinylidene difluoride membranes. The membranes were incubated with 5% milk in TBST (w/v) for 1 h and then incubated with primary antibodies diluted in 5% BSA in TBST overnight at 4 °C. Blots were incubated with horseradish peroxidase-coupled secondary antibodies (Promega, Madison, WI; R&D systems, Mckinley NE, MN) for 1 h, and protein expression was detected using SuperSignal West Pico Chemiluminescent Substrate or SuperSignal West Femto Chemiluminescent Substrate (Thermo Scientific, Waltham, MA). NIH ImageJ software was used for the quantification of blotting images. Uncropped and unprocessed scans for blots are in Supplementary Fig. 11. All blots were derived from the same experimental replicate and processed in parallel.

### RNA isolation and quantitative real-time PCR and reverse transcription PCR

Total RNA was extracted using TRIzol (Invitrogen, Carlsbad, CA) and purified using RNeasy Mini Kit (Qiagen, Hilden, Germany) and DNase I treatment. The yield and purity of RNA were estimated spectrophotometrically using the A_260_/A_280_ ratio. One µg of total RNA was reverse transcribed into cDNA using Superscript III Reverse Transcriptase (Invitrogen) and the equivalent of 10 ng was used for PCR. These reactions were carried out in a final volume of 20 μL containing 0.2 mM deoxynucleotide triphosphates, 120 nM of each primer, and 1 U Taq-DNA-polymerase. The TaqMan probes used are listed in Supplementary Table 1. Gene expression was determined by quantitative real-time PCR on an ABI Prism 7700 Sequence Detection System (Applied Biosystems). The relative RNA expression levels of target genes were analyzed by the comparative ΔΔ*C*_T_ method^33^ using *GAPDH* as an internal control, which has been reported stably expressed in all gravity conditions^34^. Subsequently, expression levels of the investigated genes were normalized to expression levels of control samples and reported as fold changes. Changes larger than 2-fold in relative mRNA expression were considered significant. The TaqMan human cyclins and cell cycle regulation gene array (Applied Biosystems; Waltham, MA) was used following the company protocol.

For reverse transcription PCR, 1 μg of total RNA was reverse transcribed using SuperScript^™^One-Step RT-PCR with Platinum^®^Taq (Invitrogen). The primer sequences for *Ki67* are for forward: TTGTGCCTTCACTTCCACAT and for the reverse: CTGGTAATGCACACTCCACCT, while for *TBP are* forward: CTCCCACCCAAAGTCTGATGA and reverse: GCCATAAACCAAGCAGGACG. The cDNA synthesis and pre-denaturation were carried out at 95 °C for 2 min. PCR amplification was performed for 35 cycles at 95 °C for 30 s, 55 °C for 30 s, and 72 °C for 30 s. The final extension cycle was run at 72 °C for 10 min. Finally, 14 μL of PCR product was loaded onto a 1.0% agarose gel. Band densitometry analysis was performed using ImageLab 6.0 (Bio-Rad, Hercules, CA, USA).

### RNA sequencing and data analysis

Total RNA (>500 ng with RIN >7.0) was used to prepare TruSeq Stranded mRNA library using the TruSeq Stranded mRNA LT Sample Prep Kit following the manufacturer’s library preparation protocol (TruSeq Stranded mRNA Sample Preparation Guide, Part #15031047 Rev. E). Whole transcriptome sequencing for six samples (three sμg and three matched control samples) was performed using Illumina NovaSeq6000 S4 sequencer. More than 100 million 2 × 151 pair-end reads were generated per sample with a Phred quality score Q30 > 90%. In order to test the robustness of the RNAseq gene expression quantification results with respect to different bioinformatics pipelines, we generated five versions of raw counts matrices using several pipelines with different parameter settings. First, RNAseq reads were aligned to GRCh38.p13 (GENCODE release 36, www.gencodegenes.org/human) using STAR (2.7.7a) with default parameters. In addition, we changed the default parameters *outFilterScoreMinOverLread*and *outFilterMatchNMinOverLread* from 0.66 to 0.30 and generated a second-version of alignment. Both RSEM (v1.3.3) and Salmon (1.4.0) were used to quantify the gene expression with their default parameters using the two versions of STAR alignments, resulting in four versions of the gene expression raw count matrices. In addition, Salmon was used in mapping-based mode (without using STAR alignments) to generate the fifth version of gene expression raw count matrices. The fifth version of gene expression results was highly consistent with each other (Supplementary Fig. 6). DESeq2 (1.30.0) was used to perform differential gene expression analysis using all five versions of the gene expression results. GSEA (v4.1.0) was used to perform gene set enrichment analysis.

### Statistical analysis

All experiments were performed at least in triplicate and data from all different cell lines was pooled for analysis, as no differences were found between them. The data were expressed as mean value ± SEM and analyzed by an unpaired *t* test. Levels of statistical significance were set at *p* < 0.05 (in the text ‘*’ means *p* < 0.05, ‘**’ means *p* < 0.005).

### Reporting summary

Further information on research design is available in the Nature Research Reporting Summary linked to this article.

## Results

### Simulated microgravity enhances self-renewal of hPSCs

A fast-rotating 2-D clinostat was developed following a previous report to generate simulated microgravity (sµg) conditions for culturing adherent cells^27^, and it was modified for the use of two Clipmax chamber slides set back-to-back. Cells were seeded on the cell culture area in the chamber slides, which is located in close proximity to the rotation axis, to expose cells to minimal centripetal forces and experience simulated chronic free fall, and where the magnitude of microgravity simulation nears zero^27^ (Fig. 1). We observed differences in hPSCs responses to sµg under 0.06g compared to 0.03g (data not shown) and performed our experiments at 0.06g.

**Fig. 2:**
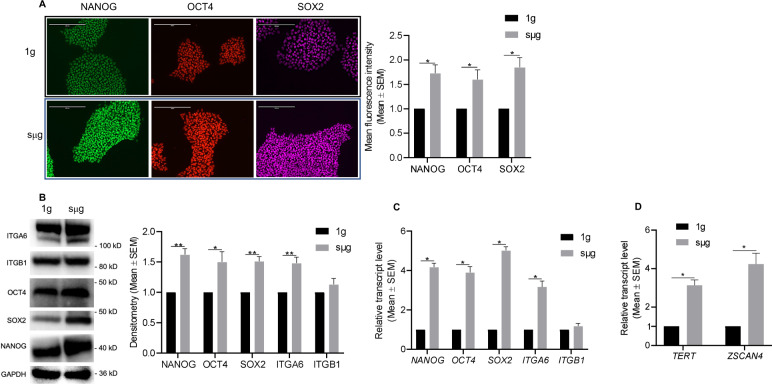
Simulated microgravity (sμg) enhances the self-renewal of hPSCs. Culture under sμg maintained self-renewal of hPSCs and enhanced the expression of the core set of pluripotent transcription factors (TF), transmembrane glycoproteins ITGA6 and ITGB1, and telomere genes. **A** Representative immunofluorescent micrographs of colonies from sμg and 1g conditions stained for the core set of pluripotent TFs. Scale bars, 200 µm. Graphs show the mean fluorescence intensity analysis from micrographs. **B** Representative immunoblots and densitometry analysis from protein lysates of cells cultured at sμg and 1g conditions. RT-qPCR analysis indicates relative mRNA levels of (**C**) pluripotent associated and (**D**) telomere elongation genes present in cells cultured at sμg and 1g conditions. *p < 0.05, **p < 0.005 (*n* = 3; unpaired *t* test). Error bars in graphs represent the SEM of the group.

The effects of simulated microgravity (sµg) on hPSCs were investigated by comparing to cells cultured in normal gravity (1g). First, we examined and confirmed that hPSCs cultured under sµg for 96 h remained undifferentiated (Fig. 2A and Supplementary Fig. 1). The expression levels of pluripotent transcription factors (TF) including the homeodomain protein NANOG, the POU domain TF OCT4 (also known as POU5F1), and the SRY-related HMG-box TF SOX2—required to establish the undifferentiated state and to maintain self-renewal of hPSCs^35–37^, and the transmembrane glycoproteins, integrin α6 (ITGA6) (also known as CD49f) and integrin β1 (ITGB1)^38,39^, were analyzed at protein and RNA levels in cells under sµg and 1g conditions. Analysis of micrographs from colonies under both conditions indicated an increase in the immunofluorescent signal of NANOG, OCT4, and SOX2 under sµg (Fig. 2A). These results were confirmed by quantification of protein bands obtained by immunoblotting (Fig. 2B). An increase in protein expression of ITGA6 was also observed, while no change was detected for ITGB1. Analysis at the RNA level indicated that there was a more than 2-fold increase in expression of human pluripotency-associated genes, including *ITGA6*, in cells under sµg in relation to 1g (Fig. 2C). It is known that the telomerase pathway is active and required for prolonged self-renewal in hPSCs^40,41^, thus, we analyzed the expression levels of *TERT* and *ZCAN4*, genes involved in telomere elongation. The results demonstrated a significant increase in their RNA expression levels in cells under sµg compared to control ones (Fig. 2D). The higher RNA levels observed during sµg returned back to basal levels once the cells were further cultured in 1g (Supplementary Fig. 2). These results indicate that sµg enhances the self-renewal of hPSCs.

**Fig. 3:**
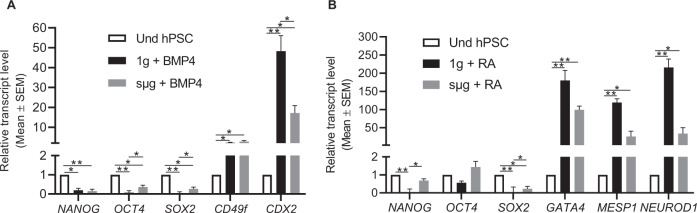
Differentiation of hPSCs is reduced under simulated microgravity (sμg) conditions. hPSCs were cultured under sμg or 1g for 48 h with a medium that sustains their self-renewal, followed by a further 48 h of culture with a differentiation medium to induce (**A**) trophectoderm and (**B**) neuroectoderm differentiation. RT-qPCR analysis of representative genes related to pluripotency, trophectoderm, and neuroectoderm indicated that differentiation under sμg condition is reduced compared to 1g condition. *p < 0.05, **p < 0.005 (*n* = 3; unpaired *t* test). Error bars in graphs represent the SEM of the group.

We analyzed the differentiation capability of hPSCs after culturing in sµg by inducing the formation of EBs and found that the cells remained pluripotent, as indicated by increased transcript-level expression of genes representative from all 3 germ layers (Supplementary Fig. 3). Due to the increased protein translation of pluripotent associated proteins in cells cultured in sµg compared to cells at 1g (Fig. 2A–C), we further tested whether their differentiation would be affected under sµg conditions. To do this, cells were first cultured in sµg with a self-renewal supporting medium for 48 h, followed by 96 h of further culturing using CDM with either BMP4 or RA to induce trophectoderm^42^ and neuroectoderm^43^ differentiation, respectively (Fig. 3). As expected, the mRNA transcript levels for the core pluripotency transcription factors (*OCT4, SOX2*, and *NANOG*) were downregulated after differentiation. However, the reduction was higher in cells cultured at 1g compared to cells at sµg. Genes related to trophectoderm and neuroectoderm showed significantly higher increases in mRNA levels in cells cultured in 1g compared to sµg (Fig. 3). These results support our previous observation that sµg conditions enhance the self-renewal of hPSCs, as their differentiation is slightly delayed or reduced compared to 1g conditions.

### Simulated microgravity increases the proliferation of hPSCs

**Fig. 4:**
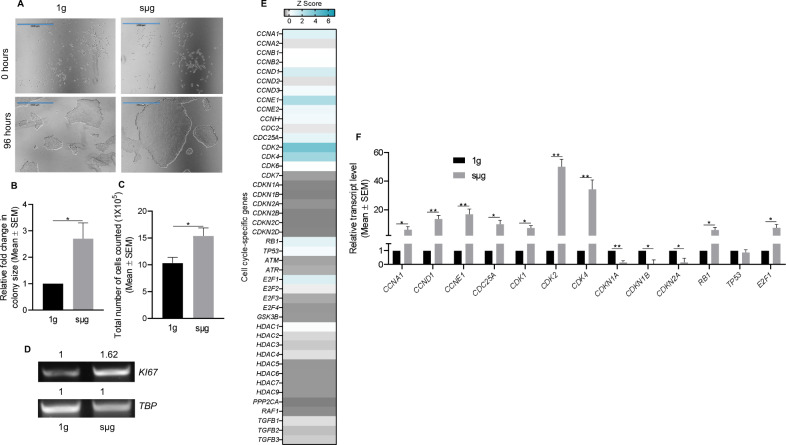
Simulated microgravity (sµg) enhances the proliferation of hPSCs. **A** Representative micrographs of hPSC colonies cultured under sμg and 1g conditions. Scale bars, 1000 µm. **B** Graphs indicating significant fold change difference in hPSC colonies area after 96 h of culture under smg and 1g conditions. **C** Graph comparing the total cell number of hPSCs after 96 h of culture under sμg and 1g conditions. **D** Representative gel band images of rt-PCR analysis for the cell proliferation gene *Ki67* in hPSCs after 96 h of culture under sμg and 1g conditions. The mRNA expression of *TBP* was used to show equal loading and to quantify the relative differences in expression, as indicated above each band. **E** Heat map showing the relative mRNA levels of 44 human cell cycle-associated genes in hPSCs culture under sμg for 96 h in relation to cells cultured in 1g condition. **F** RT-qPCR analysis was used to verify the expression of selected genes tested in the gene array assay. *p < 0.05, **p < 0.005 (*n* = 3; unpaired *t* test). Error bars in graphs represent the SEM of the group.

We observed that under sµg the colony size and the total number of cells counted per sµg chamber slides were significantly higher as compared to 1g culture condition (Fig. 4A–C and Supplementary Fig. 1). The enhanced cell proliferation of hPSCs under sµg condition was supported by increased RNA levels of the cell proliferation marker, *Ki67* (Fig. 4D). We analyzed the expression of 44 human cell cycle-associated genes using a human cell cycle regulation gene array, which revealed that multiple genes change their expression after exposure to sµg (Fig. 4E). *CDK2/4* and their cyclin counterparts, *Cyclin E* and *Cyclin D*, respectively, along with the CDK activator *CDC25A* were significantly upregulated in hPSCs cultured under sµg compared to 1g condition, whereas negative regulators were repressed in hPSCs cultured under sµg condition (Fig. 4E). These results were validated by qRT-PCR analysis, which confirmed that *CDK2/4* and its respective counterparts were significantly upregulated in hPSCs cultured under sµg condition compared to 1g culture condition (Fig. 4F). The upregulated levels of cell cycle-associated genes returned to levels comparable to cells under 1g condition after these cells were further cultured in 1g condition (Supplementary Fig. 4). To investigate whether the effects of sµg observed in hPSCs would be replicated in somatic cells, we cultured equal numbers of human foreskin fibroblasts (hFF) in sµg and 1g conditions for 96 h. The cell proliferation was not affected by sµg, as indicated by similar cell doubling time, and there were no differences in expression of *Ki67* between hFFs cultured in sµg and 1g conditions (Supplementary Fig. 5). Similarly, the expression of telomerase-related genes in hFFs was not affected by sµg (Supplementary Fig. 5), indicating a cell type-dependent effect of sµg.

### Effects of simulated microgravity on hPSC signaling pathways

**Fig. 5:**
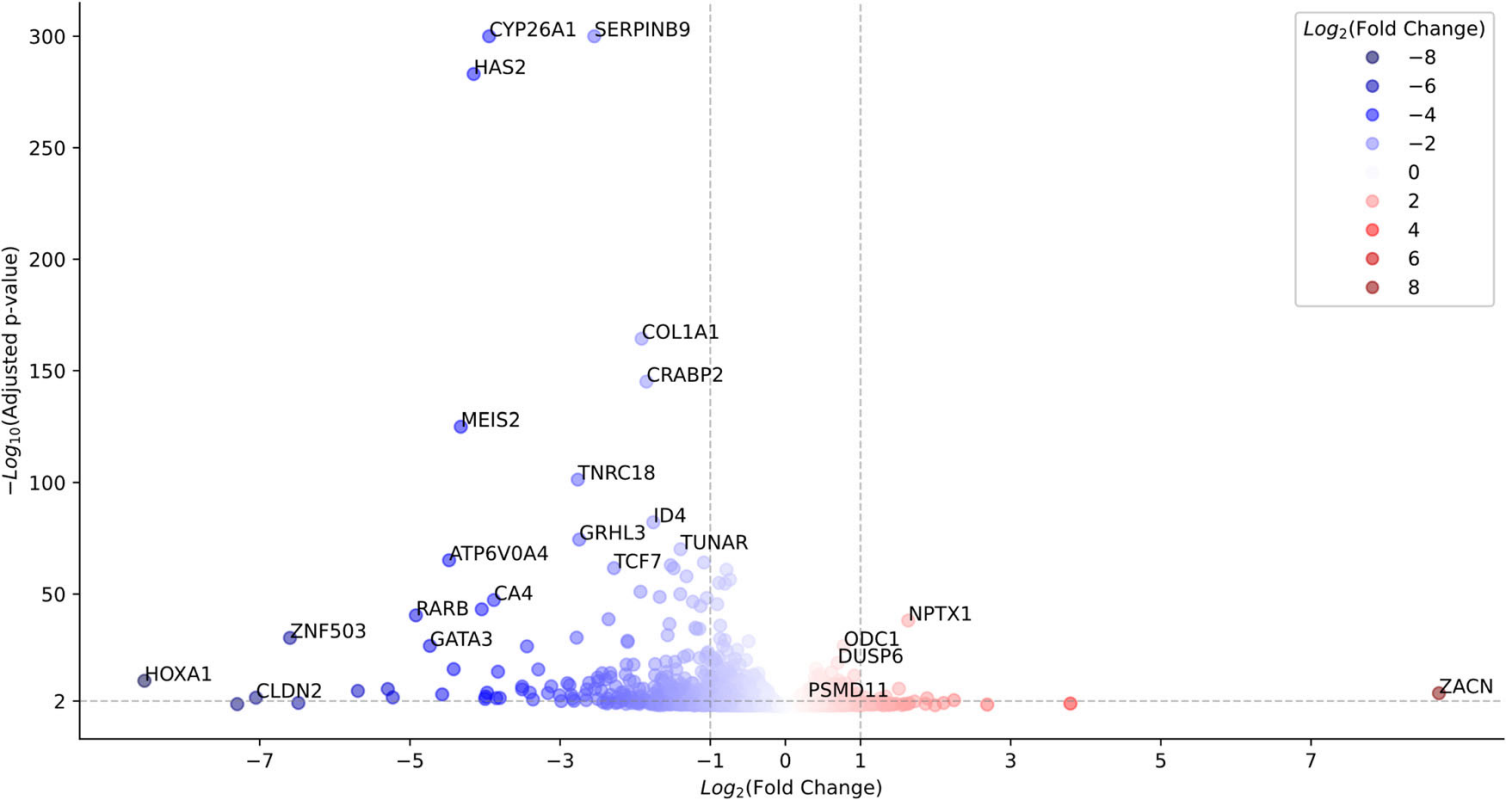
Volcano plot of differential gene expression between hPSCs cultured in simulated microgravity (sµg) and 1g condition. The color intensity is proportional to Log2 (Fold Change). Genes are overly expressed under sµg condition if Log2 (Fold Change) > 0 (colored red in the figure). The vertical dashed lines correspond to Log2 (Fold Change) = ±1, and the horizontal line corresponds to the adjusted *p*-value = 0.01. There are a few genes that are highly expressed under 1g conditions such as HAS2, CYP26A1, SERPINB9, etc.

**Fig. 6:**
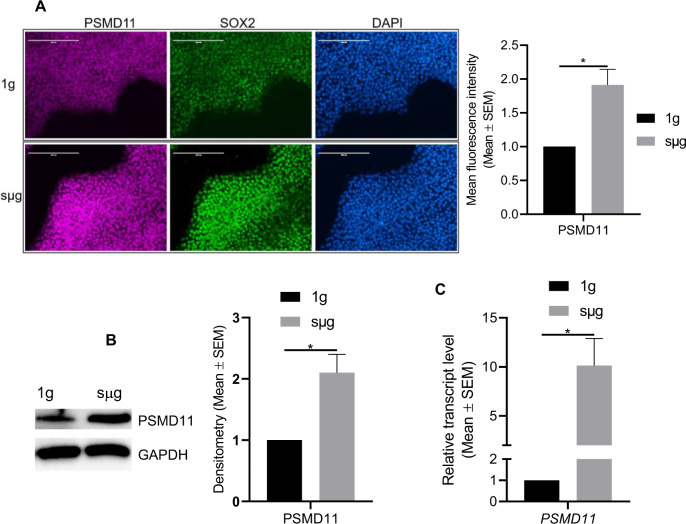
Simulated microgravity (sµg) enhances PSMD11 expression in hPSCs. **A** Representative immunofluorescent micrographs of hPSCs cultured in sμg and 1g conditions and stained for PSMD11 and SOX2. DAPI was used to stain the nuclei of all cells. Scale bars, 200 µm. Graphs showing the mean fluorescence intensity analysis from micrographs. **B** Representative Immunoblots and densitometry analysis of PSMD11 from protein lysates of cells cultured at sμg and 1g conditions. GAPDH was used as a loading control. Graphs on the right show the densitometry analysis of the immunoblots. **C** RT-qPCR analysis indicating the relative mRNA levels of *PSMD11* present in cells cultured at sμg compared to 1g condition. *p < 0.05 (*n* = 3; unpaired *t* test). Error bars in graphs represent the SEM of the group.

RNA sequencing analysis of hPSCs cultured in sµg and 1g conditions identified 1741 differentially expressed (DE) genes (FDR < 0.05) detected by the first four versions of the results (Supplementary Fig. 6). After the intersection of these 1741 DE genes with the ones detected by Salmon analysis without alignment, 1649 genes DF were found by all five versions of gene expression results. From this common set of 1649 DE genes, 626 were over-expressed and 1023 were under-expressed in the sµg group compared to the 1g group (Supplementary Fig. 7). An unbiased gene set enrichment analysis using all available Reactome pathways indicated that 532 and 677 pathways were upregulated in the sµg and 1g groups, respectively (Supplementary Tables 2, 3). Consistently with our results (Figs. 2 and 4), it was observed that pathways related to telomere maintenance and cell cycle were significantly upregulated in the sµg group compared to the 1g group, while in the 1g group pathways related to differentiation and development were upregulated (Supplementary Fig. 8). Accordingly, among the top 20 differentially expressed genes between sµg and the 1g group were genes related to development and differentiation, such as *CYP26A1, HAS2, CRABP2, MEIS2, ID4, GRHL3*, and *TUNAR*, which were downregulated in the sµg group (Fig. 5 and Supplementary Fig. 9). The analysis also showed that in 98 of the 532 upregulated pathways in the sµg group the ubiquitin-proteasome system was present (Supplementary Table 4). In particular, *PSMA1, 3*, and *7*, and *PSMB5* from the core-20S particle, all six components of the base-19S regulatory particle (*PSMC1*–*6*), as well as *PSMD11* and *PSMD14* from the lid-19S regulatory particle were upregulated (Supplementary Table 3). Real-time PCR analysis validated the upregulation of *PSMD11* in the sµg group compared to 1g condition (Fig. 6A), while an increase in PSMD11 protein translation in the sµg group was verified by ICC and WB analysis (Fig. 6B, C).

## Discussion

To our knowledge, this is the first study that has investigated the self-renewal properties of hPSCs under sµg culture conditions using a fast-rotating 2-D clinostat at 80 RPM, and demonstrated that the proliferation and self-renewal of adhered hPSCs are enhanced under sµg compared to cells in 1g condition. This was accompanied by a modulated increase in protein expression of pluripotent TFs and integrin α6, as well as by upregulation of telomerase activity and cell cycle-associated genes. The RNAseq analysis revealed that under sµg the expression of pathways related to differentiation and development were downregulated, while multiple components of the ubiquitin-proteasome system were upregulated, contributing to the enhanced self-renewal phenotype observed.

There are reports indicating that microgravity simulated by ground-based experiments may not have the same cellular effects as space flight conditions. Han et al.^44^ reported that the genes associated with proliferation and survival were upregulated in mice neural crest stem cells exposed to real space, whereas cells exposed to ground-based sµg upregulated genes associated with differentiation and inflammation. However, other studies indicate similar effects on cells exposed to zero gravity and microgravity. It has been shown that spaceflight preserves the stemness of mESC-derived progenitors and inhibits the expression of markers of terminal differentiation for tissues derived from the three primary germ layers^8^. Similar implications have been reported under simulated microgravity. For instance, it has been reported that the need for a feeder layer, serum, and Leukemia-Inhibitory Factor (LIF) in the conventional method to prevent mESCs from spontaneously differentiating was eliminated by culturing in a 3-D clinostat culture system that generates multi-directional G force, resulting in an environment with an average of 10^−3^ G^10^. Other studies of mESCs under sµg have shown varied outcomes including decreased cell numbers associated with increased apoptosis, altered adhesion properties, and differentiation^45^. Here, we are reporting that sµg has cell-type-specific effects as observed between hPSCs and hFFs. Whether the effects observed on hPSCs under sµg are replicable in zero gravity remains to be determined.

Our study with hPSCs following 96 h of cultivation under sµg and in adhering conditions, uncovered that cells remain undifferentiated and that their self-renewal is enhanced compared to 1g culture conditions, as revealed by the increased protein levels of the core set of pluripotent TFs. Blaber et al.^8^ also reported that multiple markers indicative of self-renewal of stem cells were increased under sµg. We previously identified integrin α6 (also known as CD49f and ITGA6) as a key regulator in the self-renewal of hPSCs^38^ and as the only common stem cell-related protein expressed in all 35 identified stem cell types^39,46^. Here, we also observed that sµg upregulates the expression of pluripotent TFs and integrin α6 both at gene and protein levels in hPSCs in comparison to the 1g control group. Further, our data indicate induction of telomere elongation in hPSCs cultured under sµg condition compared to 1g culture condition, supported by the mRNA upregulation of *TERT* and *ZSCAN4*, which are known to contribute to telomere elongation^40,41,47^. All of these findings indicate that sµg culture condition enhances the self-renewal of hPSCs.

Our study also demonstrated that hPSCs cultured under sµg condition remained pluripotent after returning to 1g conditions, as shown by trilineage differentiation profiling of the cells. However, the induced differentiation experiments towards trophectoderm and neuroectoderm during sµg showed that the reduction of pluripotent TFs was less evident in cells compared to 1g. The mRNA expression levels for trophectoderm and neuroectoderm markers were significantly lower in cells under sµg culture conditions when compared to 1g. All of these suggest that sµg reduces the susceptibility of hPSCs towards differentiation; supporting our conclusion that sµg enhances their self-renewal.

PSC persist in a state of rapid proliferation and they have a unique short cell cycle, which in part serves to impede differentiation^48–51^. We have shown that cell proliferation of hPSCs is increased under sµg culture conditions. We found significantly larger colonies and a high total number of cells under sµg compared to 1g culture condition and this was accompanied by significantly higher expression levels of *Ki67,* a gene related to cell proliferation, indicating that cells under sµg have a shorter cell cycle compared to cells at 1g condition (Fig. 4). This was validated by results from a qRT-PCR based microarray study showing significant upregulation of *cyclins B1, E1*, and *D1* along with their partner CDKs (*CDK1, CDK2*, and *CDK4*, respectively) under sµg compared to 1g. In addition, sµg downregulated the expression of cyclin-dependent kinase inhibitor genes (INK and CIP/KIP family of inhibitors). Analysis of RNA-seq data also indicated the upregulation of multiple cell cycle-related pathways in hPSCs cultured on sµg conditions compared to the 1g group (Supplementary Fig. 8). All of these findings suggest that under sµg conditions hPSCs have shortened cell cycle and this may be supported by the enhanced self-renewal and reduced susceptibility towards differentiation of hPSCs, as reported previously^50,52–54^. We observed that the sµg effects on hPSCs were restricted and reversible to the culture condition, as the proliferation rate returned to comparable levels of hPSCs cultured in 1g condition after cells were further expanded at 1g conditions (Supplementary Fig. 5). Interestingly, the effects of sµg on cell proliferation and telomerase activity were cell-type dependent as were not replicated in hFFs (Supplementary Fig. 5).

Using RNA-sequencing analysis, we identified a significant number of DEG in sµg when compared to 1g. Functional analysis of these genes and overlap of GO terms indicated that cells grown under sµg condition displayed enrichment of biological process GO terms, including mRNA processing, mRNA stability, translation, regulation of transcription, chromatin organization, and telomerase maintenance, all of which align well with our results discussed above and might be responsible for the enhanced proliferation and self-renewal observed under sµg. Multiple members of the ubiquitin-proteasome system were upregulated under sµg culture condition, and we verified the upregulation at RNA and protein level of PSMD11 under sµg (Fig. 6). This indicates that the proteolytic activity of the proteasome complex, 26S/30S proteasome, is enhanced under sµg. It has been established that both mESCs and hPSCs exhibit increased levels of proteasome activity and assembly of the 26S/30S proteasomes^55,56^. This upregulation has been linked to increased levels of the 19S proteasome subunit PSMD11/RPN-6^55,57^, an essential subunit for the assembly and activity of the 26S/30S proteasome^58^. It is known that in hPSCs, PSMD11 maintains self-renewal by protecting the expression of pluripotency markers and by targeting the degradation of germ layer specific markers^55^. In addition, it has been shown that differentiation of hPSCs results in a downregulated expression of PSMD11/RPN6 with subsequently reduced proteasomal activity and a reduction in the amount of assembled proteasome complexes. The downregulated expression of PSMD11/RPN6 during differentiation is accompanied by a decrease in hydrolytic activity of the proteasome complex, suggesting that PSMD11/RPN6 is essential for preserving the activity of the proteasome. Based on this information and our results, we concluded that under sµg conditions the ubiquitin proteasome system is participating in the enhanced self-renewal of hPSCs.

Pertaining to our results and other previous reports regarding the crosstalk between stem cell and cell cycle machineries^35,38,48,50,59^, we have developed a model explaining the regulation of proliferation and self-renewal of hPSCs by sµg (Supplementary Fig. 10). The canonical model highlights the interplay between the core set of pluripotent TFs, including OCT4, SOX2, and NANOG, and a common transmembrane glycoprotein heterodimeric complex formed by ITGA6 and ITGB1, for maintaining proliferation and self-renewal of hPSCs by interacting with major regulators of the cell cycle. Under sµg condition, our model shows that hPSCs enhanced their proliferation and self-renewal by overexpression of the core set of pluripotent TF complex and glycoprotein heterodimeric complex signaling networks (both represented in brown color in our model). In addition, the ubiquitin-proteasome system (represented by PSDM11) is also upregulated (represented in brown color), providing further support to the self-renewal machinery. In turn, the core set of pluripotent TF complex may upregulate the expression of major cell cycle regulators (represented in green) maintaining a shorter cell cycle and reducing the susceptibility towards differentiation, resulting in an enhanced self-renewal of hPSCs. These findings are critical to both space science and cell biology due to its potential to decipher the effect of microgravity on humans at a cellular level as well as in the regeneration of tissues in the human body and to use these alterations to better understand and solve spaceflight-based problems.

## Supplementary information

Supplementary Information Final

Reporting Summary

## Data Availability

All the data generated or analyzed during this study are included in this published article, if not, are available from the corresponding author on request. The RNA-seq data is available in the GEO repository GSE205559.
