# Supplementary material for: Enhanced self-renewal of human pluripotent stem cells by simulated microgravity
Source: NPJ Microgravity. 2022 Jul 4;8:22. doi: 10.1038/s41526-022-00209-4 (PMC9253108; doi:10.1038/s41526-022-00209-4)
Supplement: Supplementary file 1 — Supplementary Information Final [file 41526_2022_209_MOESM1_ESM.pdf]

Supplementary Information

Enhanced self-renewal of human pluripotent stem cells by simulated microgravity

Timilsina, S.,[1] Kirsch-Mangu, T.,[1] Werth, S.,[3] Shepard, B.,[1] Ma, T.,[2] Villa-Diaz, L.G.[1,3,*]

Department of Biological Sciences[1], Department of Computer Science and Engineering[2], and Department of Bioengineering[3], Oakland University, Rochester, MI 48309

Funding support:

This research was supported with funding from the Michigan Space Grant Consortium, Oakland University REF funding, and the NSF grant 2026049.

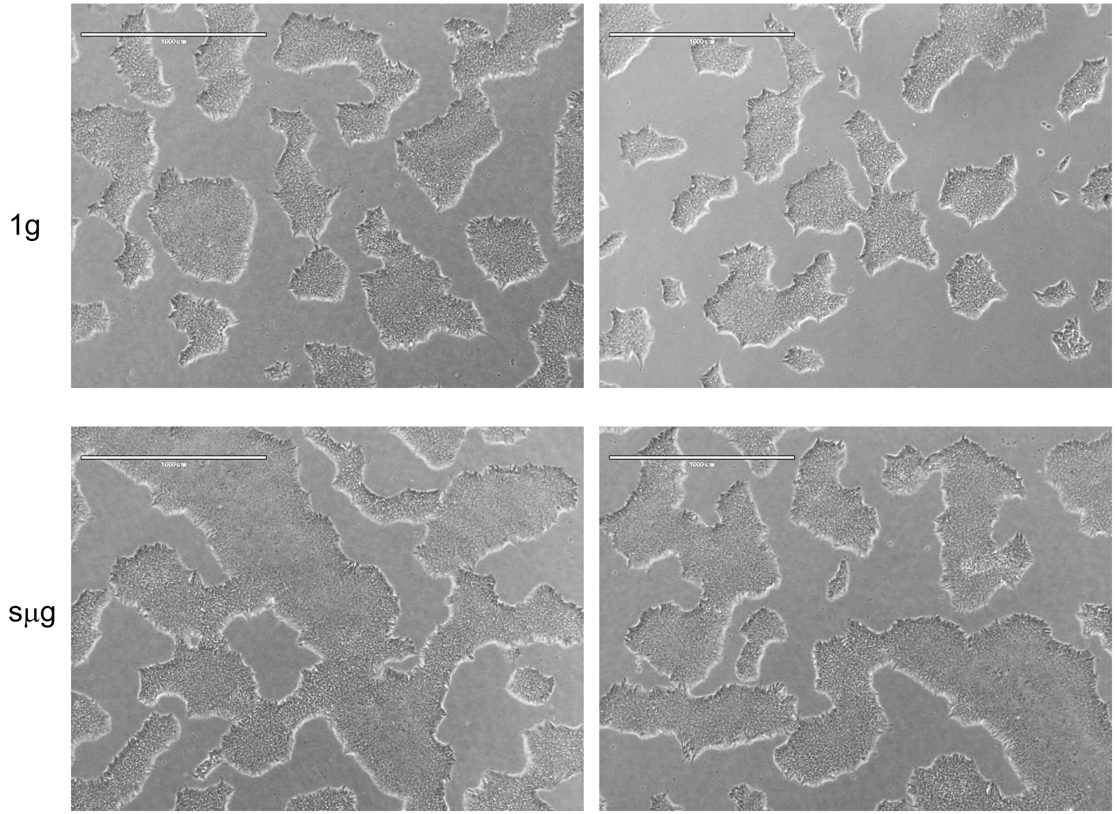

Supplementary Figure 1. Culture under simulated microgravity (smg) conditions enhances cell proliferation of hPSC. Representative micrographs of colonies from smg and 1g conditions after 96 h of culture. Equal number of cells were initiated seeded for both conditions. Scale bars, 1000 μm.

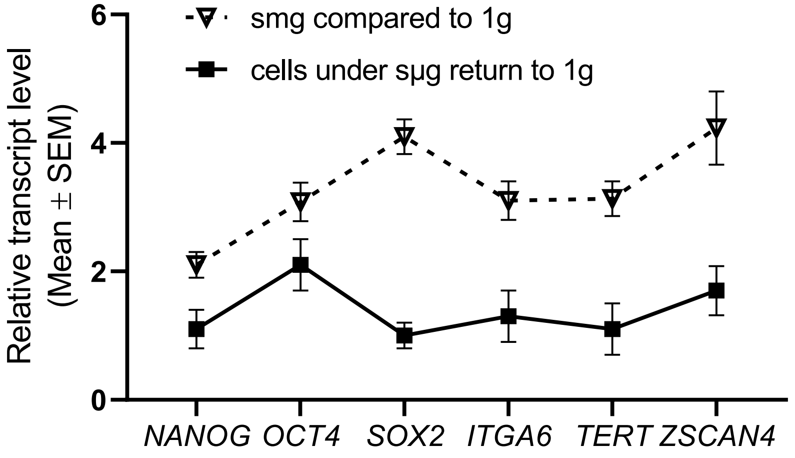

Supplementary Figure 2. Effects of simulated microgravity (sμg) on hPSC are reversible. RT-qPCR analysis indicating relative mRNA levels of pluripotent associated and telomere elongation genes in cells initially cultured under sμg and with additional culture in 1g condition for additional 48 hours. Error bars in the graph represent the SEM of the group.

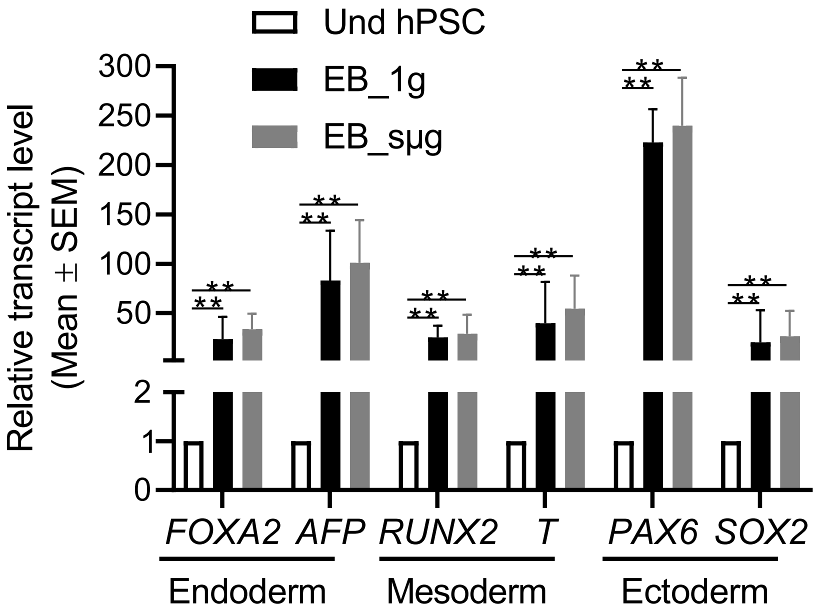

Supplementary Figure 3. hPSC cultured in simulated microgravity (sμg) conditions remain pluripotent. RT-qPCR analysis of genes for 3 germ layers from EB made from cells cultured under sμg and 1g compared to undifferentiated hPSC. ** $p < .005$ (n=3), by unpaired *t*-test. Error bars in the graph represent the SEM of the group.

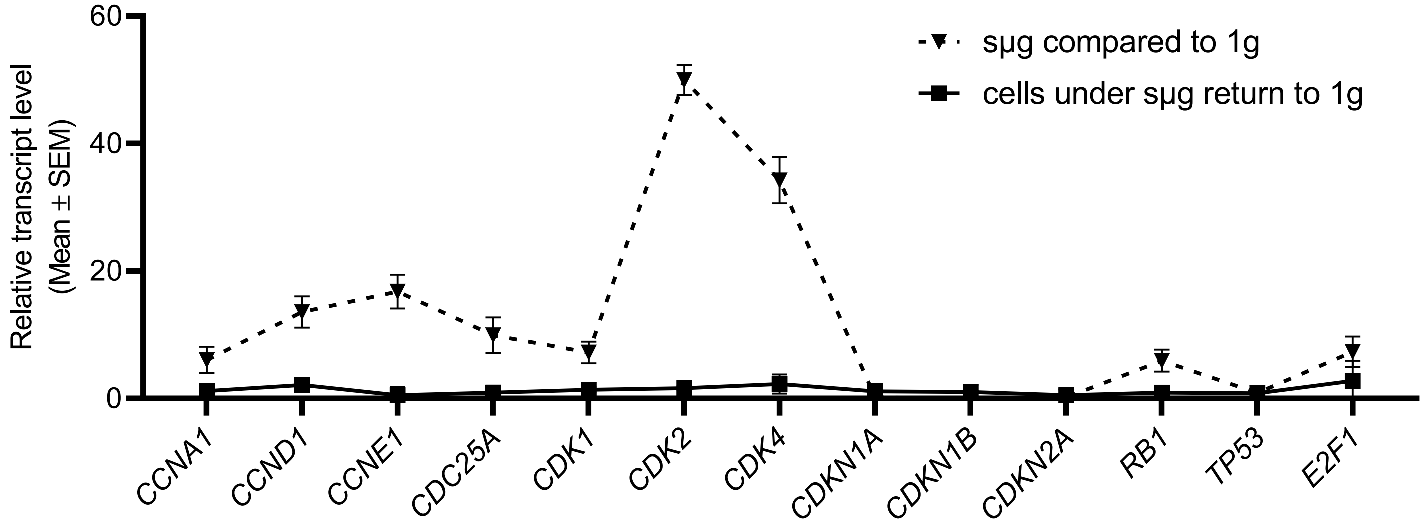

Supplementary Figure 4. The effect of simulated microgravity (sμg) in cell proliferation of hPSC is reversible. RT-qPCR analysis showing the return of high expressing of cell cycle associated genes from hPSC cultured under sμg for 96 h to levels expressed in cells cultured in 1g condition, after further cultured in 1g condition for 48 hours. Error bars in the graph represent the SEM of the group.

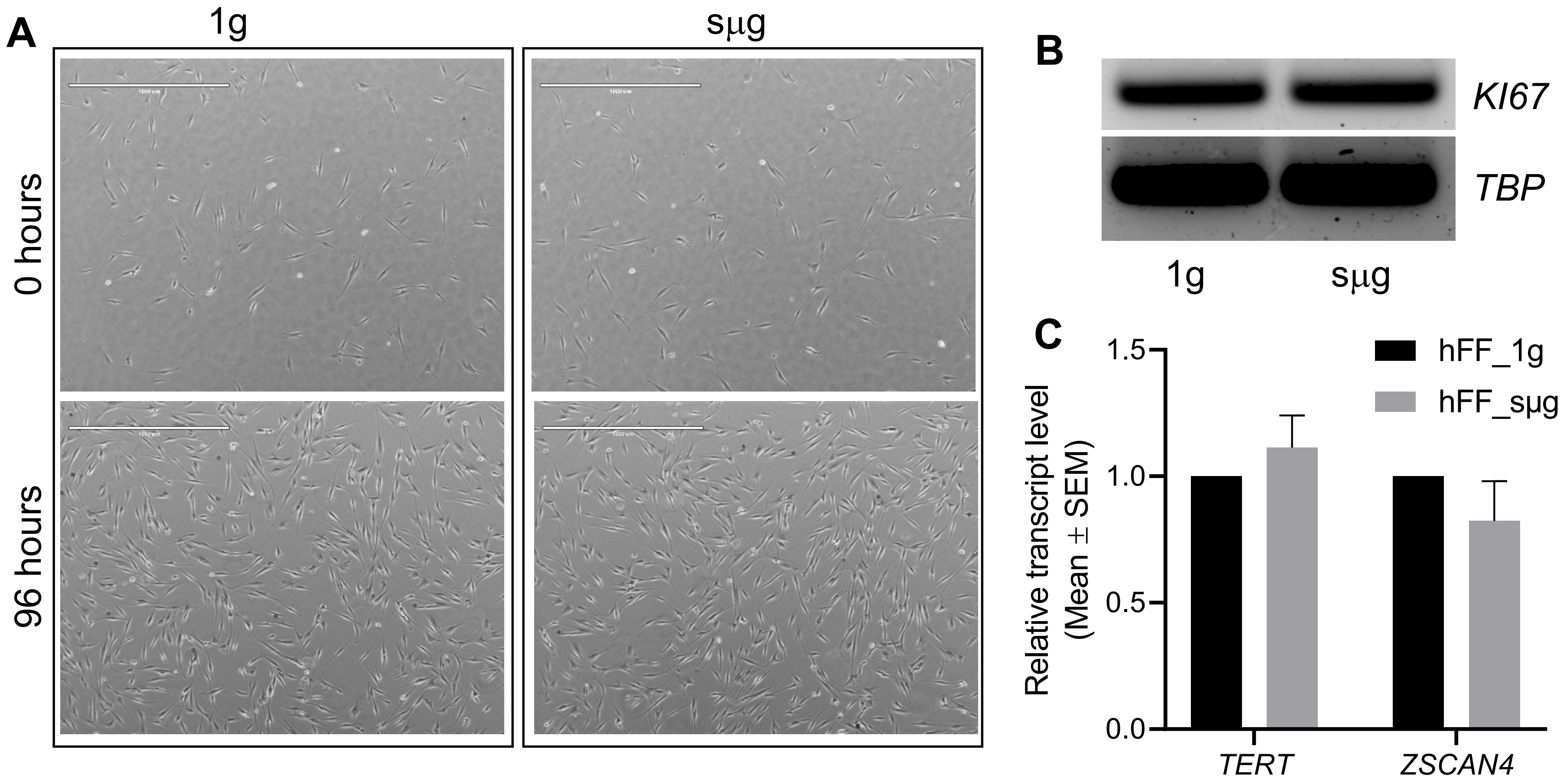

Supplementary Figure 5. Somatic cells are not affected by simulated microgravity (sμg) as hPSC. Human foreskin fibroblasts (hFF) were cultured under sμg and 1g, and the results indicated that are not affected as hPSC. The cell proliferation is not affected by sμg as indicated by (**a**) representative micrographs after 96 h and (**b**) the relative mRNA expression of *KI67* in hFF cultured under sμg and 1g conditions. (c) RT-qPCR analysis indicating the relative mRNA levels of telomere elongation genes in hFF cultured under sμg and compared to 1g condition. Scale bars, 1000 μm. Error bars in the graph represent the SEM of the group.

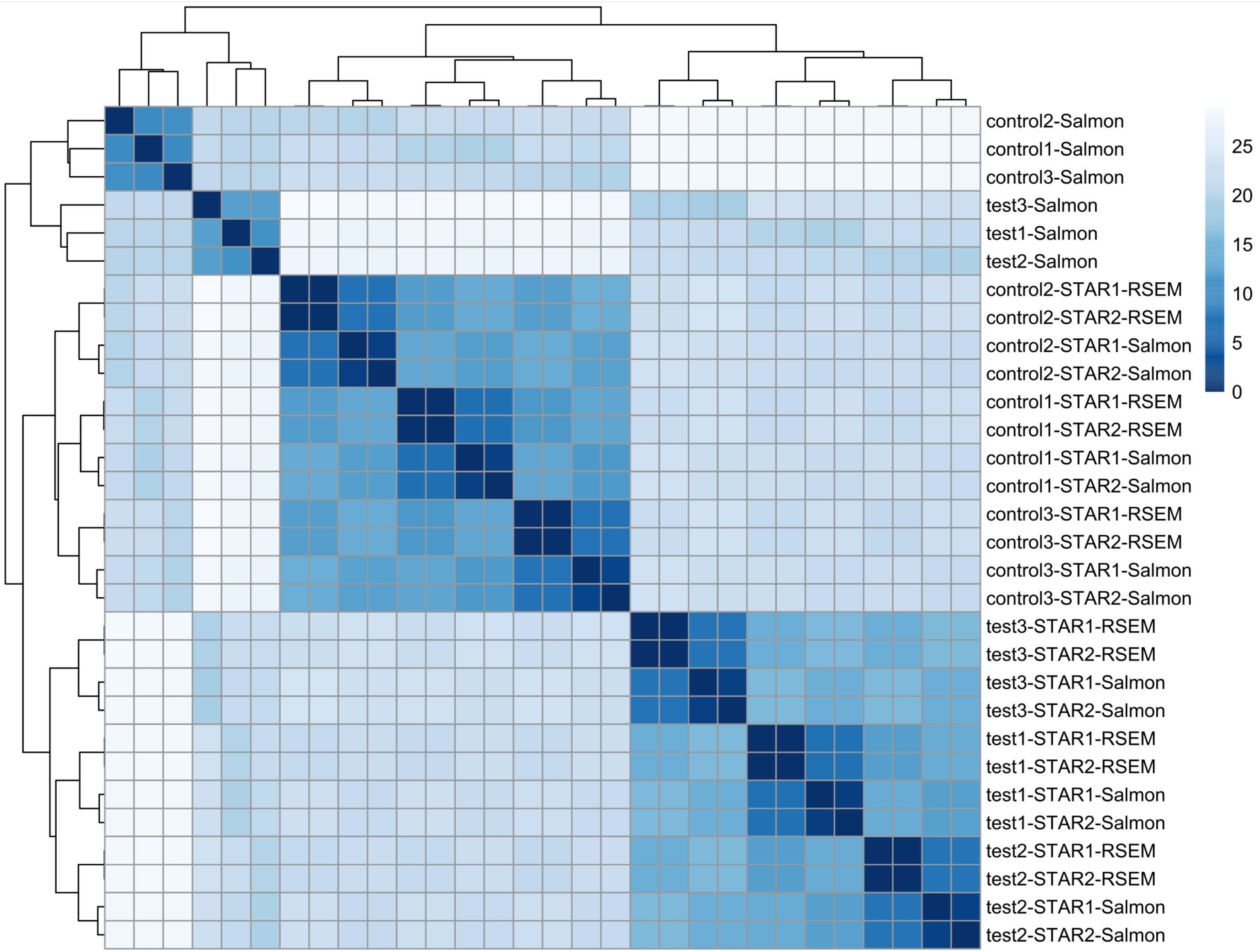

Supplementary Figure 6. Heat map of sample distances based on five versions of normalized counts. Five versions of gene expression normalized counts are named STAR1-RSEM, STAR2-RSEM, STAR1-Salmon, STAR2-Salmon, and Salmon, which are the suffixes of the sample names. The prefixes of sample names are control1-3 and test1-3, corresponding to three samples under 1g and smg conditions, respectively. Gene expression quantification using Salmon alone is considered less accurate, while the other four versions of gene expression results are highly consistent with each other.

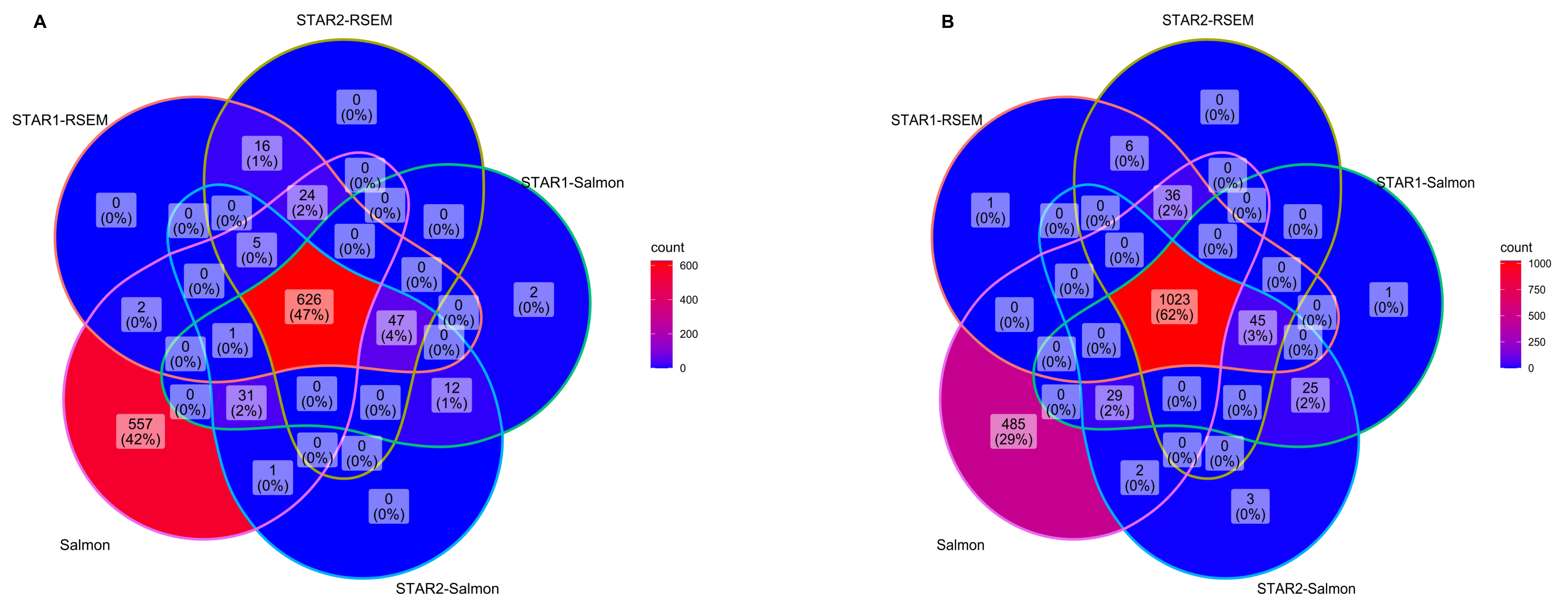

Supplementary Figure 7. Venn diagrams of differentially expressed genes based on five versions of gene expression results. Five versions of gene expression normalized counts are named STAR1-RSEM, STAR2-RSEM, STAR1-Salmon, STAR2-Salmon, and Salmon. **a** Over-expressed genes detected under smg condition, **b** under-expressed genes under smg condition. Gene expression quantification using Salmon alone is considered less accurate, while the other four versions of gene expression results are highly consistent with each other.

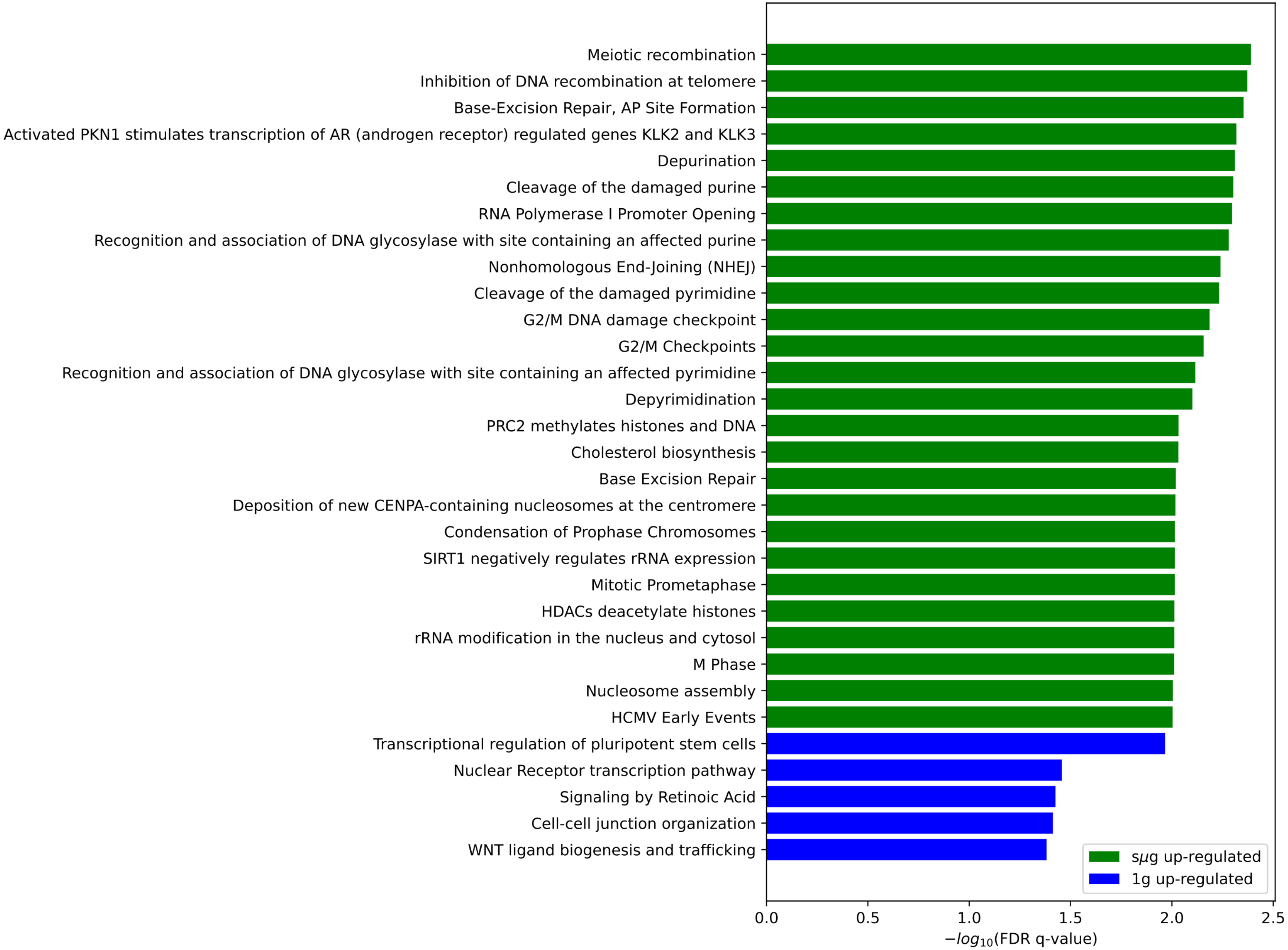

Supplementary Figure 8. Bar plot of top up-regulated pathways under sμg and 1g conditions.

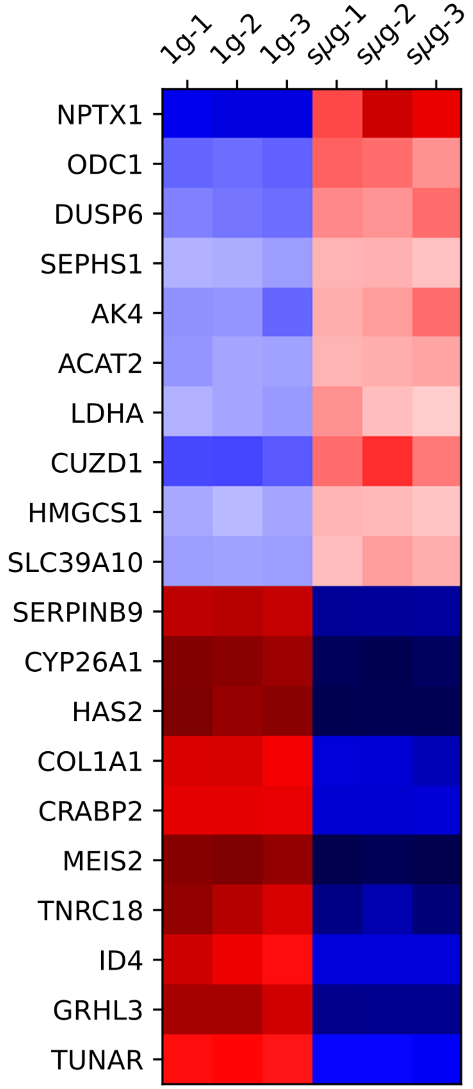

Supplementary Figure 9. Heat map of top 20 differentially expressed genes between simulated microgravity (sμg) and 1g conditions. The color intensity is proportional to relative expression levels with red corresponding to higher expression levels.

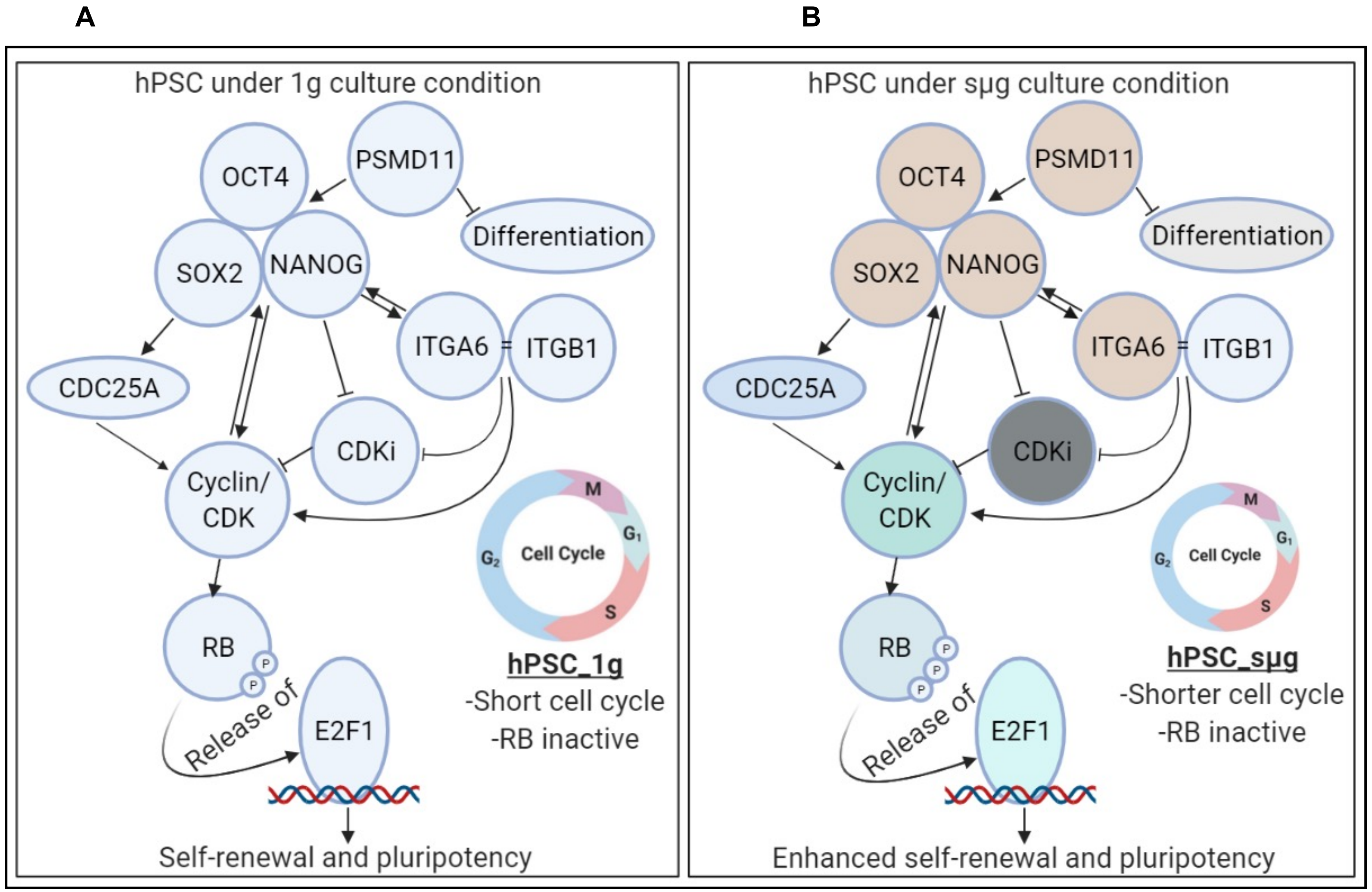

Supplementary Figure 10. Schematic model of mechanisms behind the enhanced self-renewal and proliferation of hPSC cultured with simulated microgravity (sμg). **a** Under 1g conditions the interplay between the core set of pluripotent transcription factors (TF: OCT4, SOX2, and NANOG), and the transmembrane glycoprotein heterodimeric complex formed by integrin a6 (ITGA6) and integrin b1 (ITGB1) maintain proliferation and self-renewal of hPSC by interacting with major regulators of the cell cycle. **b** The smg model shows that hPSC's enhanced expression of PSMD11, suggesting an increased ubiquitinase activity targeting differentiation related genes, while protecting the core pluripotent TF (represented in brown color). This in turn upregulates (represented in green color) the expression of the major cell cycle regulators, while suppresses negative regulators of cell cycle progression (represented in grey color), resulting in shorter cell cycle, enhanced proliferation and maintaining prolonged self-renewal.

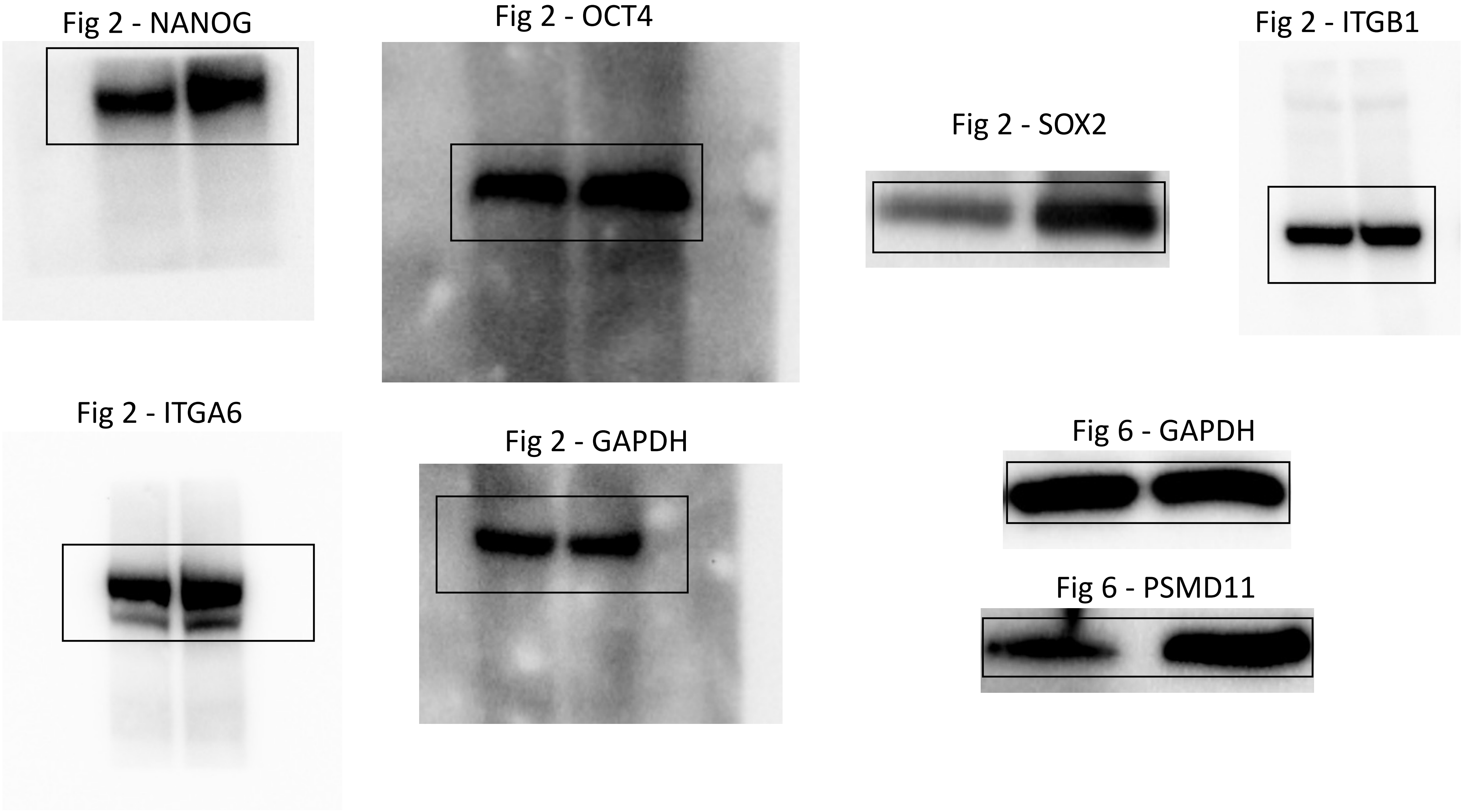

Supplementary Figure 11. Unprocessed blots as obtained by ChemiDoc™ Touch Imaging System.
