# Supplementary material for: Enhanced self-renewal of human pluripotent stem cells by simulated microgravity
Source: NPJ Microgravity. 2022 Jul 4;8:22. doi: 10.1038/s41526-022-00209-4 (PMC9253108; doi:10.1038/s41526-022-00209-4)
Supplement: Supplementary file 2 — Reporting Summary [file 41526_2022_209_MOESM2_ESM.pdf]

nature portfolio

Corresponding author(s): Villa-Diaz, LG

Last updated by author(s): 03/02/2022

Reporting Summary

Nature Portfolio wishes to improve the reproducibility of the work that we publish. This form provides structure for consistency and transparency in reporting. For further information on Nature Portfolio policies, see our Editorial Policies and the Editorial Policy Checklist.

Statistics

For all statistical analyses, confirm that the following items are present in the figure legend, table legend, main text, or Methods section.

n/a	Confirmed	
☐	☒	The exact sample size (*n*) for each experimental group/condition, given as a discrete number and unit of measurement
☐	☒	A statement on whether measurements were taken from distinct samples or whether the same sample was measured repeatedly
☐	☒	The statistical test(s) used AND whether they are one- or two-sided<br>*Only common tests should be described solely by name; describe more complex techniques in the Methods section.*
☒	☐	A description of all covariates tested
☒	☐	A description of any assumptions or corrections, such as tests of normality and adjustment for multiple comparisons
☐	☒	A full description of the statistical parameters including central tendency (e.g. means) or other basic estimates (e.g. regression coefficient) AND variation (e.g. standard deviation) or associated estimates of uncertainty (e.g. confidence intervals)
☐	☒	For null hypothesis testing, the test statistic (e.g. *F*, *t*, *r*) with confidence intervals, effect sizes, degrees of freedom and *P* value noted<br>*Give P values as exact values whenever suitable.*
☒	☐	For Bayesian analysis, information on the choice of priors and Markov chain Monte Carlo settings
☒	☐	For hierarchical and complex designs, identification of the appropriate level for tests and full reporting of outcomes
☒	☐	Estimates of effect sizes (e.g. Cohen's *d*, Pearson's *r*), indicating how they were calculated

Our web collection on statistics for biologists contains articles on many of the points above.

Software and code

Policy information about availability of computer code

Data collection	GENCODE release 36, www.gencodegenes.org/human using STAR (2.7.7a) with default parameters. In addition, we changed the default parameters outFilterScoreMinOverLreadand outFilterMatchNMinOverLread from 0.66 to 0.30 and generated a second-version of alignment. Both RSEM (v1.3.3) and Salmon (1.4.0) were used to quantify the gene expression with their default parameters using the two versions of STAR alignments, resulting in four versions of the gene expression raw count matrices. In addition, Salmon was used in mapping-based mode (without using STAR alignments) to generate the fifth version of gene expression raw count matrices. GSEA (v4.1.0) was used to perform gene set enrichment analysis.
Data analysis	GraphPad InStat and GraphPad Prims were used.

For manuscripts utilizing custom algorithms or software that are central to the research but not yet described in published literature, software must be made available to editors and reviewers. We strongly encourage code deposition in a community repository (e.g. GitHub). See the Nature Portfolio guidelines for submitting code & software for further information.

Data

Policy information about availability of data

All manuscripts must include a data availability statement. This statement should provide the following information, where applicable:

- Accession codes, unique identifiers, or web links for publicly available datasets
- A description of any restrictions on data availability
- For clinical datasets or third party data, please ensure that the statement adheres to our policy

All the data generated or analyzed during this study are included in this published article, if not, are available from the corresponding author on request.

Field-specific reporting

Please select the one below that is the best fit for your research. If you are not sure, read the appropriate sections before making your selection.

☒ Life sciences ☐ Behavioural & social sciences ☐ Ecological, evolutionary & environmental sciences

For a reference copy of the document with all sections, see nature.com/documents/nr-reporting-summary-flat.pdf

Life sciences study design

All studies must disclose on these points even when the disclosure is negative.

Sample size	All experiments were performed at least in triplicates with the different cell lines.
Data exclusions	No data was excluded from the analysis.
Replication	The experiments were replicated by multiple investigators/authors, showing consistent and replicable results.
Randomization	Cells for individual experiments were obtained from a common source and distributed randomly to the experimental groups.
Blinding	No blinding experiments were performed, however, all the experiments contained appropriate internal controls

Reporting for specific materials, systems and methods

We require information from authors about some types of materials, experimental systems and methods used in many studies. Here, indicate whether each material, system or method listed is relevant to your study. If you are not sure if a list item applies to your research, read the appropriate section before selecting a response.

Materials & experimental systems

n/a	Involved in the study	
☐	☒	Antibodies
☐	☒	Eukaryotic cell lines
☒	☐	Palaeontology and archaeology
☒	☐	Animals and other organisms
☒	☐	Human research participants
☒	☐	Clinical data
☒	☐	Dual use research of concern

Methods

n/a	Involved in the study	
☒	☐	ChIP-seq
☒	☐	Flow cytometry
☒	☐	MRI-based neuroimaging

Antibodies

Antibodies used	OCT4 (Santa Cruz Biotechnology), NANOG (Cell Signaling Technology), SOX2 (Cell Signaling Technology), Integrin α6 (Millipore), Integrin β1 (Santa Cruz Biotechnology), PSMD11 (Novus Biologicals; Centennial, CO) and GAPDH (Cell Signaling Technology).
Validation	All antibodies were validated by molecular weight, and protein target immunolocalization in specific cell lines, in addition to the information provided by vendor.

Eukaryotic cell lines

Policy information about cell lines

Cell line source(s)	NIH-approved human embryonic stem cell lines H1 and H9 (WiCell Research Institute Inc., Madison, WI) and human foreskin fibroblasts from ATCC.
Authentication	The cell lines were authenticated by the information provided by WiCell Research Institute and ATCC.
Mycoplasma contamination	All cell lines were tested for mycoplasm contamination before use in experiments and were negative.
Commonly misidentified lines (See ICLAC register)	*Name any commonly misidentified cell lines used in the study and provide a rationale for their use.*
